# Identification of Potential Small Molecule Binding Pockets on Rho Family GTPases

**DOI:** 10.1371/journal.pone.0040809

**Published:** 2012-07-16

**Authors:** Juan Manuel Ortiz-Sanchez, Sara E. Nichols, Jacqueline Sayyah, Joan Heller Brown, J. Andrew McCammon, Barry J. Grant

**Affiliations:** 1 Department of Chemistry and Biochemistry and Center for Theoretical Biological Physics, University of California San Diego, La Jolla, California, United States of America; 2 Department of Pharmacology, University of California San Diego, La Jolla, California, United States of America; 3 Howard Hughes Medical Institute, University of California San Diego, La Jolla, California, United States of America; 4 Department of Computational Medicine and Bioinformatics, University of Michigan, Ann Arbor, Michigan, United States of America; MRC National Institute for Medical Research, United Kingdom

## Abstract

Rho GTPases are conformational switches that control a wide variety of signaling pathways critical for eukaryotic cell development and proliferation. They represent attractive targets for drug design as their aberrant function and deregulated activity is associated with many human diseases including cancer. Extensive high-resolution structures (>100) and recent mutagenesis studies have laid the foundation for the design of new structure-based chemotherapeutic strategies. Although the inhibition of Rho signaling with drug-like compounds is an active area of current research, very little attention has been devoted to directly inhibiting Rho by targeting potential allosteric non-nucleotide binding sites. By avoiding the nucleotide binding site, compounds may minimize the potential for undesirable off-target interactions with other ubiquitous GTP and ATP binding proteins. Here we describe the application of molecular dynamics simulations, principal component analysis, sequence conservation analysis, and ensemble small-molecule fragment mapping to provide an extensive mapping of potential small-molecule binding pockets on Rho family members. Characterized sites include novel pockets in the vicinity of the conformationaly responsive switch regions as well as distal sites that appear to be related to the conformations of the nucleotide binding region. Furthermore the use of accelerated molecular dynamics simulation, an advanced sampling method that extends the accessible time-scale of conventional simulations, is found to enhance the characterization of novel binding sites when conformational changes are important for the protein mechanism.

## Introduction

**Figure 1:**
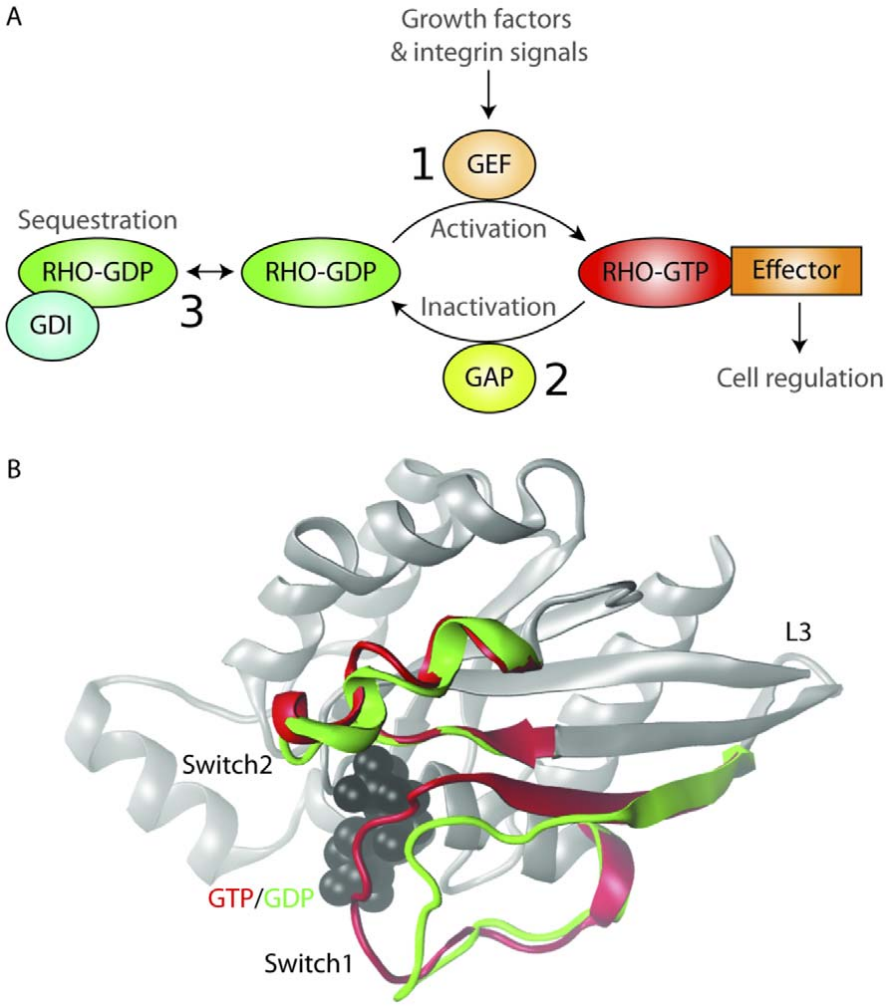
Rho GTPase activation. (A) Schematic representation of the Rho GTPase activation cycle. (1) The GTP-bound active state is generated by guanine exchange factors (GEFs), promoting the exchange of GDP for GTP. (2) GTPases activating proteins (GAPs) catalyze the hydrolysis of GTP to GDP, restoring the GDP-bound inactive state. (3) Stabilization of the GDP-bound inactive state by GDP dissociation inhibitors (GDIs). (B) The molecular structure of representative the GTP and GDP-bound Rho conformations (PDB codes: 1KMQ and 1FTN). The switch regions of the GTP-bound form are shown in red and GDP-bound form in green.

Rho proteins are eukaryotic intracellular signaling hubs. They function to relay signals from cell-surface receptors to signaling cascades that control diverse cell processes including gene transcription, [1] cell-cycle progression, [2], [3] and cytoskeleton reorganization. [4] Like other members of the Ras GTPase superfamily, Rho proteins act as conformational switches, hydrolytically cycling between active GTP-bound and inactive GDP-bound conformations. Three major classes of regulatory proteins modulate Rho’s activity: guanine nucleotide exchange factors (GEFs), GTPase-activating proteins (GAPs) and guanine nucleotide dissociation inhibitors (GDIs) (**Figure 1A**). [5] Association with GEFs promotes Rho activation by stimulating the exchange of GDP for GTP. This exchange of nucleotide induces important conformational changes in structural regions termed switch 1 (residues 24–40) and switch 2 (residues 57–75), (see **Figure 1B**). [6], [7] This change in conformation allows active Rho to interact with a variety of protein effectors that initiate a network of signals affecting cell functions. Regeneration of the inactive form of Rho is promoted by the action of GAPs, which stimulate GTP hydrolysis and formation of the inactive GDP conformation. The third class of regulatory proteins, GDIs can sequester inactive GDP-bound Rho proteins and prevent their activation. Disturbing the balance of these GEFs, GAPs and GDIs can affect the fidelity of the activation cycle and have severe consequences for the wide variety of cellular events that are orchestrated by Rho dependent signaling.

Deregulated Rho activity can induce aberrant phenotypes that have been linked to the initiation and progression of multiple cancers [8], [9] as well as cardiovascular [10] and neurological disorders. [11] For example, the upregulation of RhoA is observed in breast, colon, lung, gastric bladder and testicular cancer. [12], [13] In addition, the overexpression of RhoC, Rac1, Rac2, Rac3 and Cdc42 have been observed in a number of cancers. [9], [14] Rearrangement of the RhoH gene that leads to a defective cycling between GDP- and GTP-bound forms is associated with non-Hodgkin’s lymphomas. [15], [16] Furthermore, the role of Rho family proteins in the expression of NF-kB-dependent genes and the migration of leukocytes along with their interaction with the Angiotensin II pathway indicate that therapeutically targeting Rho proteins may also have applications in the treatment of inflammatory and cardiovascular diseases. [17], [18].

Targeting drugs to Rho GTPases and related signaling pathway members is believed to have significant therapeutic potential. [10], [19], [20], [21], [22], [23] Inhibitory mutants of RhoA, RhoG, Rac1 and Cdc42 prevent Ras transformation of fibroblasts, and activated mutants of these proteins are transforming. [14] Furthermore, anti-RhoA and anti-RhoC siRNAs have been shown to inhibit the growth and angiogenesis of tumors in mouse models [24]. Existing strategies for targeting deregulated Rho signaling include inhibitors of geranylgeranylation and other post-translational modifications of Rho [25], [26], [27]. These compounds have the potential to attenuate C-terminal lipid modifications required for plasma membrane localization and subsequent signaling. A drawback of such inhibitors is their poor selectivity as they likely affect many lipid-modified proteins. Another approach involves inhibitors of downstream Rho effectors such as Rho kinase (ROCK). Several ROCK inhibitors have been successful in preclinical studies, highlighting the potential benefit of clinical Rho pathway inhibition. [28], [29], [30], [31] However, because Rho proteins utilize a multitude of downstream effectors, a particular effector inhibitor will likely impair only a subset of Rho malfunctions leading to potentially limited therapeutic benefits. The design of small molecule inhibitors that directly block the nucleotide binding site (NBS) of Rho has thus far been complicated by the conserved nature of this site throughout the larger Ras superfamily. Compounds that interact with this site have a very high likelihood of unspecifically blocking other important G-protein mediated pathways.

Currently very little attention has been placed on targeting distal non-nucleotide binding sites that may offer the possibility of modulating Rho activity in a more selective and specific manner. We have recently proposed [32] that conformational selection in Ras like G-proteins including Rho is the dominant mechanism underlying the nucleotide-dependent conformational cycle. [33] Additionally, simulations have predicted allosteric coupling of the NBS with the membrane interacting C-terminus in both Rho and Ras. In agreement with our predictions, Buhrman and collaborators described allosteric modulation of Ras by mutation experiments of residues distal to the NBS. [34] Building on these findings we developed a multi-level computational approach to discover inhibitors of Ras. This approach combined novel pocket identification, conventional molecular simulations, ensemble docking and experimental testing of computationally selected inhibitors. [35] Of particular note, cell-based assays confirmed that a number of the chosen distal binding site directed compounds could inhibit the downstream signaling activity of Ras. Here we build and expand on this approach to exhaustively characterize potential small molecule binding sites on Rho.

Although many binding site search algorithms have been developed, [36] the predictions obtained with these algorithms are often specific to the single structure they were applied to. Here we employ a combination of over one hundred available Rho crystallographic structures and advanced molecular dynamics simulations to provide a more complete mapping of potential binding sites in all major conformational states. Conventional and accelerated [37] molecular dynamics simulations were used together for the first time in the structure-based characterization of binding sites. These simulations allowed us to identify less frequently visited Rho conformations, including those intermediate between GTP and GDP states. Both fragment and grid based mapping algorithms were employed to locate binding sites on both crystal and simulated structures. Our results reveal the presence of novel binding pockets in two relevant regions of Rho: close to the residues responsible for the GTP-GDP switching mechanism but outside the nucleotide binding site, and more distal sites that are allostericaly linked to the nucleotide and effector binding site regions. Some of these novel sites are more accessible in the GDP-bound (inactive) state of Rho, and present a potential novel mechanism of inhibition. Furthermore, we show that the advanced sampling technique, accelerated molecular dynamics, can be used to enhance sampling of binding sites particularly when important conformational changes are intrinsic to the target protein activity.

## Results and Discussion

### Crystallographic Structure Analysis

**Figure 2:**
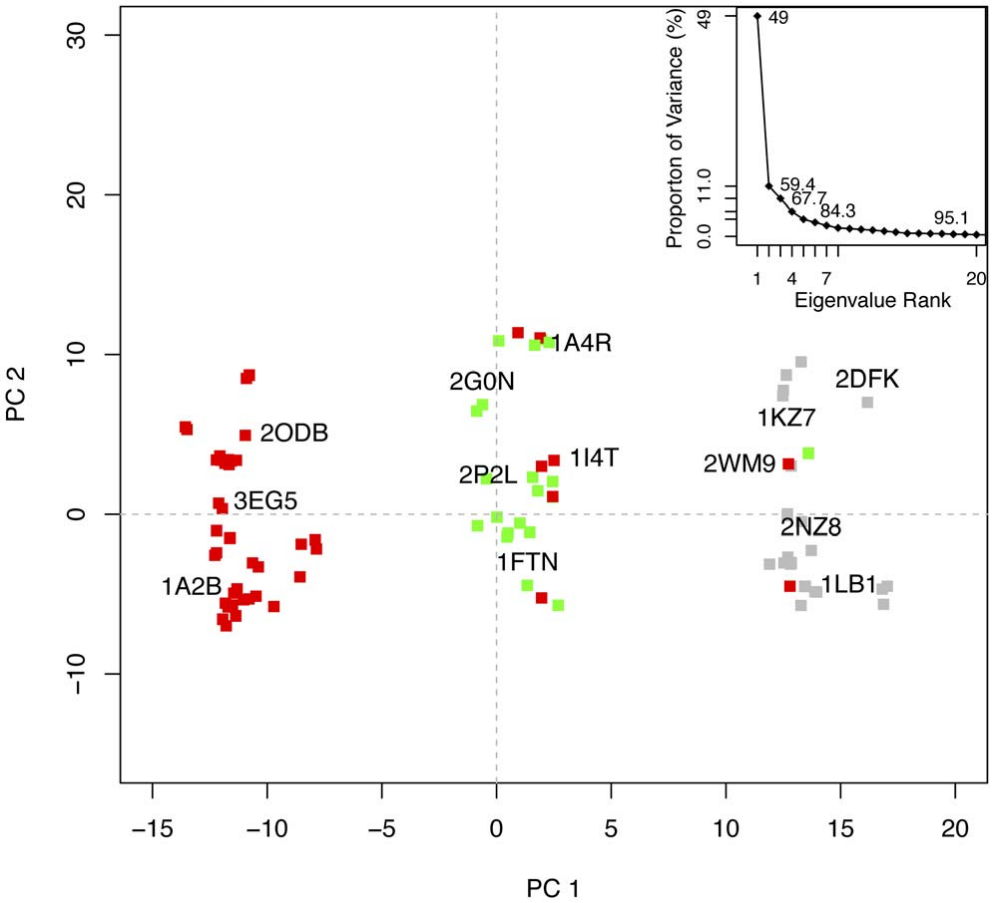
Results of PCA on Rho crystal structures. Conformer plot of available Rho GTPase crystal structures projected in the planes defined by the two most significant principal components (termed PC1 and PC2, see insert). Crystallographic GTP structures are colored red, GDP green, and nucleotide free gray. Inset: Eigenvalue spectrum: results obtained from diagonalisation of the covariance matrix of aligned atom coordinates from the Rho crystal structures. The magnitude of each eigenvalue is expressed as the percentage of the total variance (mean square fluctuation) captured by the corresponding eigenvector. Labels indicate the cumulative sum of the proportion of the total variance accounted for in all preceding eigenvectors.

High resolution structural data for the Rho family is particularly rich, given the current availability of 59 crystallographic structures comprising 98 distinct chains in the RCSB Protein Data Bank (date of access 02/10/2011). [38] These structures (see **Table S1**) span the three major nucleotide bound states (with GTP, GDP and nucleotide free). We employed principal component analysis (PCA) to examine the interconformer relationships within this crystallographic dataset (see Methods). Over 80% of the total mean-square displacement (or variance) of atom positional fluctuations was captured in six dimensions, 59.4% in two dimensions and 67.7% in three dimensions (see **Figure 2**). The first few principal components retain most of the variance in the original distribution and thus provide a useful description of the conformational space of the system. **Figure 2** depicts the projection of all Rho crystallographic structures onto the principal planes defined by the two most significant principal components. The first principal component is particularly informative and indicates the existence of three major conformationally distinct categories. These categories, or conformational clusters, are largely consistent with the nature of the bound nucleotide in each structure and correlate with the results of clustering based on pairwise RMSD values (**Figure S1**). Note that there are some GTP bound structures (including PDB codes 2ATX, 2GCO and 2WMO) that most closely resemble GDP-bound (central green points) or nucleotide free (gray) structures. This is also evident for other structurally related nucleotide binding proteins, such as kinesin and myosin, [39] and suggests a complex underlying dynamic relationship between global conformation and nucleotide state. [39] For the purposes of this study we concluded that the ensemble of over 100 available Rho crystal structures contains significant representatives from all three nucleotide associated conformational states (42% GTP, 28% GDP and 30% APO), thus allowing us to search for potential binding sites in each major Rho conformation. Furthermore, we believe that the size and diversity of the crystallographic ensemble is sufficiently large to support our binding site analyses and comparisons to simulated conformers discussed below.

### Binding Site Mapping of Crystal Structures

**Figure 3:**
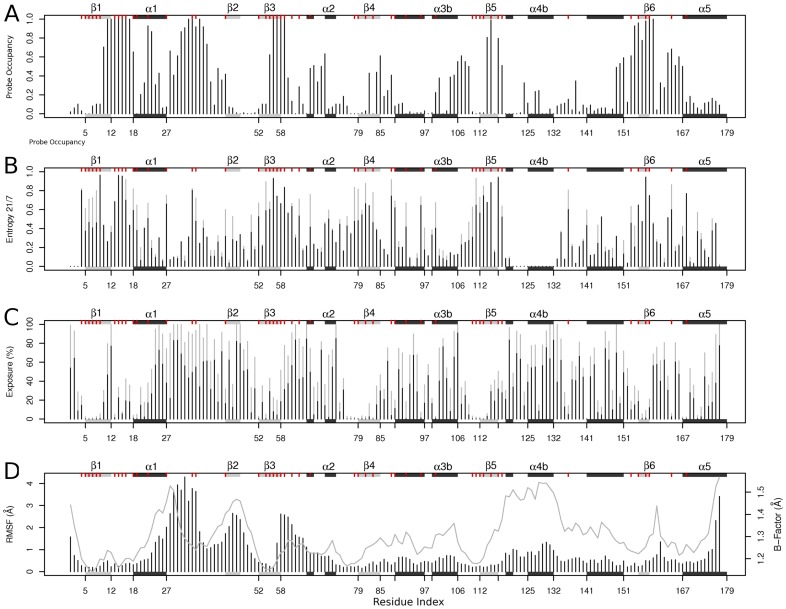
Fragment binding site characterization in the crystallographic dataset. (A) Fragment probe occupancy per position in Rho across all available structures. (B) Sequence conservation entropy scores for a 21-letter alphabet (20 amino acids and a gap, in black) and 7-letter alphabet (where amino acids are grouped into six classes based on their physicochemical properties, in light gray). (C) The mean (black) and maximum (light gray) solvent exposure per position in all structures. (D) Flexibility per position in terms of RMSF (bars) and mean *B*-factor (line) over all structures. Major elements of secondary structure (shaded rectangles) and positions with a high degree of sequence conservation (red ticks) are indicated in the marginal areas of each plot to facilitate comparison. Both the secondary structure and residue numbering are according to the RhoA-GDP complex from *Homo Sapiens* (PDB code 1FTN).

A fragment-mapping based approach (FTMAP) was used to locate potential small molecule binding hot spots on each of the Rho crystallographic structures. [40] Based on the same principle behind screens for small organic fragment binding by NMR and X-ray crystallography, FTMAP correlates pocket druggability with propensity to bind clusters of small organic compound fragments. This approach was found to outperform a number of other geometric and ligand based pocket identification methods in an earlier study. [35] To further verify mapping results for the current application, we also employed the energetic grid based SiteMap method, [41], [42] (see Methods and **Figure S2** for details). In order to characterize the location of each potential binding site the probe occupancy per position was calculated as the fraction of structures in which a given residue can coordinate a fragment. A probe occupancy value of 1 indicates the existence of a proximal binding site in all crystallographic structures. Occupancy values of 0 indicate the complete absence of a binding site in the vicinity of a particular residue. Intermediate values reveal the intrinsic dynamic nature of certain binding pockets, present in some structures but absent in others. Note that a certain fraction of these later pockets would not be identified if only a single crystal structure representative was analyzed (**Figure S3**). **Figure 3** displays the results of this analysis together with Ras superfamily wide sequence conservation, relative solvent exposure and structural variability per position (see full details in Methods). Similar binding site profiles were obtained from SiteMap analysis (correlation value of r = 0.9, see **Figure S2**).

The highest probe occupancy values were found for the known nucleotide binding site region (residues 11–17, 31–37 and 56–59 corresponding to P-loop, switch1 and switch2 regions). This result confirms the presence of this sequence conserved solvent accessible cavity in all the analyzed crystal structures (**Figure 3A–C**). The P-loop region of this pocket (residues 11–17, encompassed by secondary elements β1, loop1 and α1) presents a relatively rigid substructure (with low RMSD values) consistent with its role in coordinating the nucleotide. In contrast, the conformationally responsive switch regions (residues 24–40 and 57–75) display higher structural fluctuations and slightly lower probe occupancy values (most notably in the highly dynamic switch 1 region, residues 31–37) (**Figure 3D**) Targeting this sequence conserved binding pocket could lead to undesirable off-target effects in other GTPases.

Targeting a distal allosteric pocket may offer significant selectivity and affinity advantages for Rho GTPase drug design. The probe occupancy analysis presented in **Figure 3A** provides a general landscape for such pocket locations over all crystallographic structures. However, information about the differences between the binding site distributions in the three major conformational states of Rho is also informative. Significant differences in the binding site landscape in the GTP, GDP and APO states could present an opportunity to stabilize one nucleotide-bound state by interacting with state specific binding pocket features. To investigate this possibility, we quantified the differences in absolute probe occupancy between the three major Rho states.

**Figure 4:**
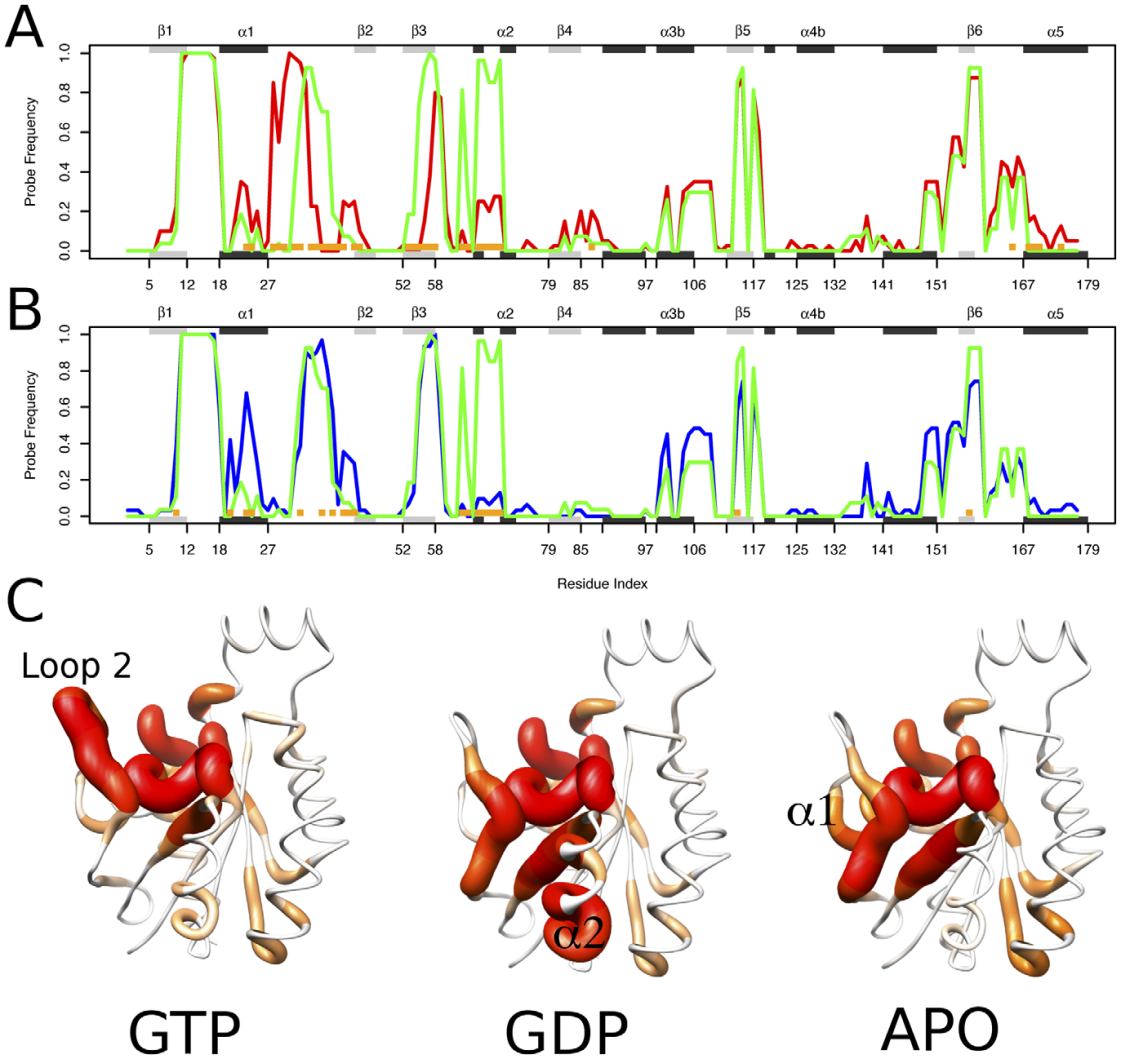
Nucleotide state associated differences in probe occupancy. (A) Comparison of the absolute probe occupancy per position between GDP/GTP and (B) GDP/APO structure sets. Probe occupancies are colored in red, green and blue for GTP, GDP and APO states. Dashed lines indicate positions in the nucleotide binding site. Orange squares indicate residues with statistically significant differences in probe occupancy (*p*<0.05) between each structure set. Major elements of secondary structure (shaded rectangles) are indicated in the marginal top areas of each plot. (C) Increasing colored radii representation of the absolute probe occupancy per residue in the GTP, GDP and APO crystal structure ensembles.

**Figure 4A–B** shows the absolute probe occupancy comparison between GTP/GDP and GDP/APO states. Orange squares along the x-axis indicate the position of residues with statistically significant differences (with a *p*-value less than 0.05). As shown, most of the significantly different regions, in terms of probe occupancy, between GTP and GDP structures lay in the NBS region. This is a useful positive control indicating the conformational differences in this pocket associated with the interconversion between the GTP and GDP forms. There is a noticeable lack of difference between GDP/APO states with respect to the previous GDP/GTP comparison. Since APO crystal structures are usually obtained by isolating Rho from the GDP/GTP exchange process, [43] after the GDP nucleotide is released (**Figure 1A**), it is reasonable that the NBS arrangement of the APO and GDP states share extensive similarities. **Figure 4C** displays probe occupancy results mapped to the molecular structure, indicating the most populated areas with an increasing red chain radii representation. Thick red regions represent high occupancy, whereas thin white segments correspond to low occupancy areas. This analysis clearly highlights the presence of distinct binding sites on each state of Rho. For example, sites centered the loop2 (GTP), α2 (GDP) and α1 (APO) regions.

Several regions exhibit a marked preference for higher probe occupancy in GDP structures than GTP and APO. The most prominent correspond to the clusters of Trp-55, Asp-56, Thr-57, Ala-58, Gly-59 and Asp-67, Thr-68, Ala-69, Gly-70, Gln-71, located in the β3 and α2 structural elements, respectively. These groups of residues lay in close proximity to loop 4 (Switch 2), one of the regulatory regions for GTP-GDP exchange in Rho. In addition, residues Thr-107, Pro-108 and Ile-109 also form a group of amino acids with significant probe occupancy differences between GTP and GDP states, and favored in the latter. These residues reside in Loop 7 close to the pocket formed by residues 149, 161 and 153 (also detected in this analysis) and are believed to be an allosteric modulator region in Ras. [34] Indeed, residues in the vicinity of these pockets are involved in correlated motions with the active site (**Figure S4**), suggesting that ligand binding at these pockets may allosterically modulate the active site architecture, as shown in other proteins. [44], [45], [46] This is consistent with the report that divalent ion-cyclen binding at an analogous pocket to that flanked by Loop 7 in Ras stabilizes a conformation that has weak effector-binding potential. [47].

In summary, the large number of available Rho crystal structures has allowed us to locate more binding pockets than could be found from analysis of individual structures. In addition, the comprehensive nature of the dataset allowed us to track how the accessibility of these pockets varies as a function of conformation, consistent with their distinct functionally relevant nucleotide state. Given the clear nucleotide related clustering of crystal structures evident in the PCA analysis and their distinct pocket properties evident from fragment mapping (**Figures 2** and **4**), it was hypothesized that additional conformations not evident in the crystallographic dataset (including those intermediate between the three major conformational states), may also be relevant for pocket determination. In order to investigate this further, and hence perform a more exhaustive search of binding sites, we have employed both conventional and accelerated molecular dynamics simulations.

### Conventional Molecular Dynamics

**Figure 5:**
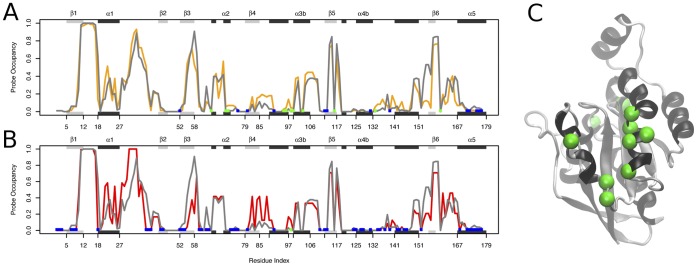
Probe binding results from conventional molecular dynamics simulations. The probe occupancy for the ensemble of crystal structures (gray) and the **A** combined conventional molecular dynamics (cMD) conformers (orange), and **B** an individual cMD GTP trajectory (red). Blue and green squares at the bottom of each plot highlight those residues that are involved in a binding pocket only in the crystal structure data set (blue) or the trajectory dataset (green). **C** Binding site positions found in cMD that are not observed in any crystallographic structure, see text for details.

We sought to investigate whether a single or multiple conventional molecular dynamics simulations (cMD) could resolve new pockets in addition to those evident in the crystallographic dataset. We first carried out three 30 ns long cMD simulations, commencing from each of the three major conformational states (PDB codes for the initial structure models are 1A2B, 1FTN and 1LB1 for the GTP GDP and APO states respectively, see **Figure 2**). Conformations from each trajectory were analyzed with the FTMap method and the probe occupancy per position computed as described previously (**Figure 5**).

**Figure 5A** depicts the probe occupancy per residue profile obtained for the combined cMD trajectory conformers (orange) together with that obtained from analysis of the complete ensemble of crystal structures (gray). Also shown in **Figure 5B** are results obtained for a single GTP initiated trajectory (red). Blue and green points at the bottom of each plot indicate residue positions found to interact directly with probe molecules in one dataset but not in the other (blue for those absent in the trajectory conformers and green for those absent in crystal structures). Clearly multiple cMD simulations are required to obtain a binding site distribution comparable to that evident in the large distribution of crystal structures (note the large number of blue points obtained when only a single trajectory is considered, **Figure 5B**). This is also evident in the higher Pearson correlation coefficient for the probe occupancy per residue between crystal structures and the combined trajectories (0.92) and obtained with individual GTP (0.84), GDP (0.82) or APO (0.89) state initiated trajectories. The combined cMD data also highlights a number of new binding site positions not evident in the crystal structures (green points in **Figure 5A**). These positions (including residues 68 to 106) were found to cluster in the three dimensional structure defining an enlarged pocket between the α2 and α3 structural elements distal to the NBS (**Figure 5C**).

**Figure 6:**
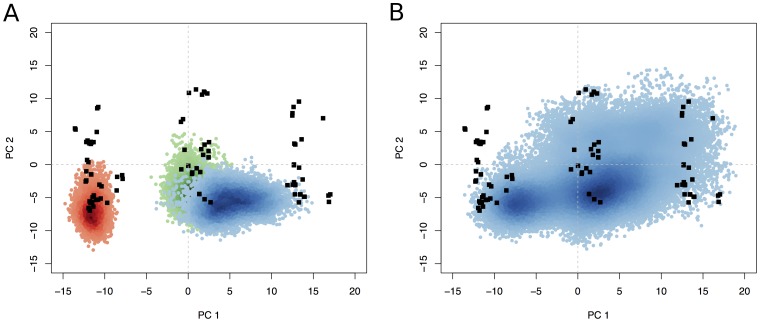
Conformational sampling in conventional and accelerated molecular dynamics simulations. The projection of (A) cMD and (B) aMD conformers onto the first two principal components of the crystallographic ensemble. The distribution of simulated conformers is depicted with density-shaded red, green and blue points for the GTP, GDP and APO simulations respectively. Crystal structures conformations are in black (see **Figure 2** and main text for details).

Principal component and RMSD analysis was used to assess the conformational space sampled in our cMD simulations and its relation to available crystal structures. **Figure 6A** shows the distribution of sampled conformers (density-shaded red, green and blue points for GTP, GDP and APO simulated systems) along with available crystal structures (black points) projected onto the dominant eigenvectors obtained from analysis of the crystal structure ensemble described previously. The GTP and GDP simulations were found to exhibit a relatively restricted sampling that is localized to regions around the corresponding cluster of crystallographic structures. The lack of overlap of these distributions is consistent with the high minimal inter trajectory RMSD value (1.33 Å) between GDP and GTP simulations). This data indicate an absence of interconversion between the GTP and GDP states under these simulation conditions. In contrast, the APO trajectory (blue points in **Figure 6A**) displays a wider sampling pattern that encompasses GDP like conformers and displays a low minimal RMSD to the GDP trajectory (0.73 Å). This is consistent with results from our previous studies of Ras where conformational sampling was observed to be more restricted in the presence of the bound nucleotide than the sampling obtained in the absence of nucleotide. [32].

In summary, individual cMD simulations did not recover the full binding site distribution evident in the large set of crystallographic structures. Multiple combined cMD simulations were more successful in mapping potential binding sites and highlighted a number of additional potential pocket interactions not recovered in the crystallographic structures. These novel potential interactions are directly related to the observation of new binding site configurations not evident in the ensemble of available crystal structures. Most notably, the cMD simulations provide information about the intermediate conformations between the GDP and APO states. The inclusion of these intermediate conformations reveals novel potential pocket interactions that are favored in the GDP/APO states. However, the current cMD simulations provide no information about conformations intermediate between GTP and GDP like states. Given the enhanced predictive performance of multiple cMD simulations and their ability to map novel pocket features, we pursued a more exhaustive conformational search by enhancing the exploration of conformational space using accelerated molecular dynamics simulations.

### Accelerated Molecular Dynamics

Accelerated MD (aMD) simulations modify the potential energy landscape explored in cMD by raising the energy minima thus lowering the effective barriers between states resulting in an enhanced degree of conformational sampling (see Methods for details). In order to test the utility of aMD simulations for pocket identification with respect to our cMD and crystallographic datasets, we performed a single 60 ns long aMD simulation, commencing from a single nucleotide free structure. Principal component analysis was again used to assess the conformational space explored by the aMD simulation (**Figure 5B** density-shaded blue points). Results indicate that the aMD simulation explores a wider region of conformational space than individual, or indeed cumulative, cMD simulations (see **Figure 5**A). In addition, the aMD simulation provides details on the intermediate regions between all three major conformational states of Rho.

**Figure 7:**
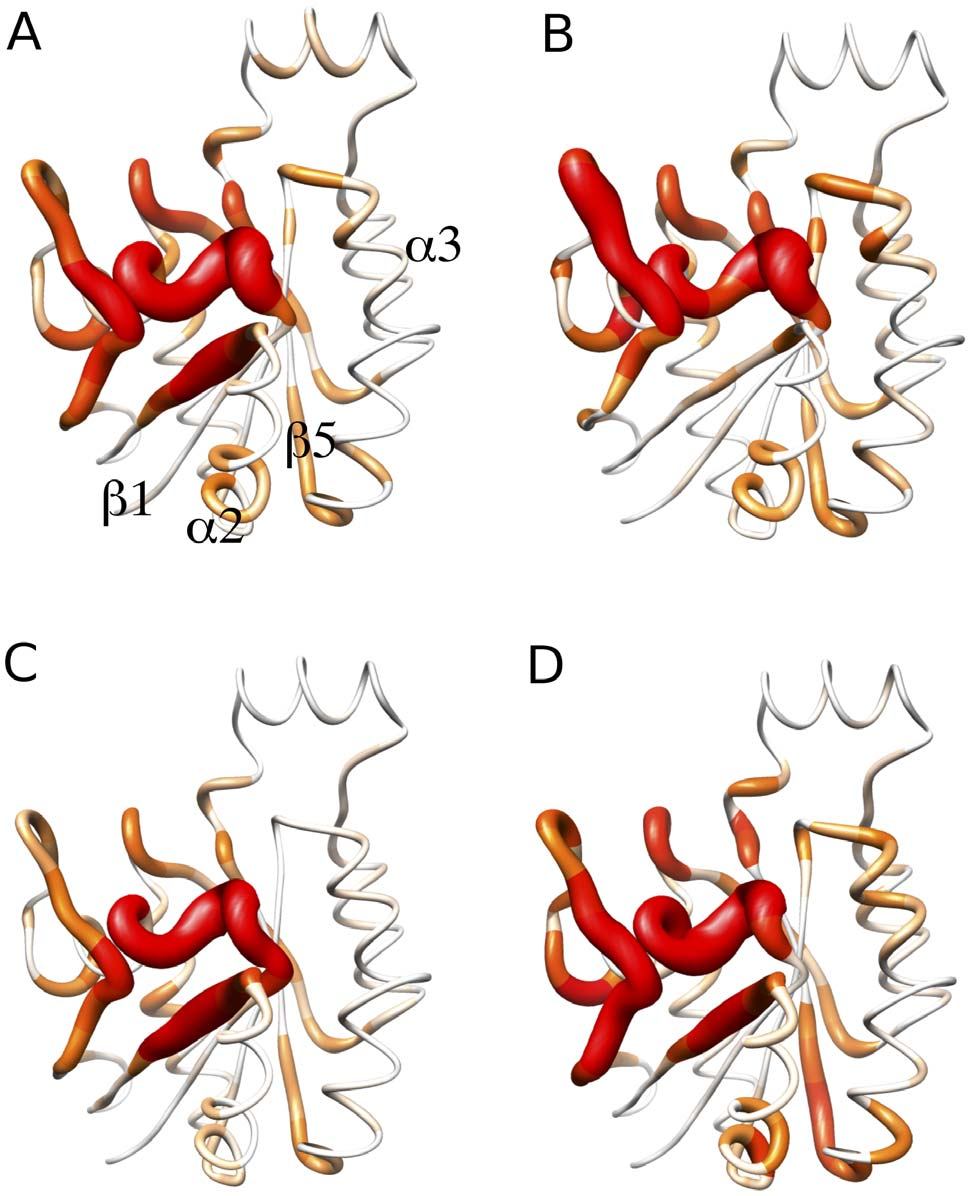
Absolute probe occupancy results from fragment mapping analysis. (A) results from the ensemble of crystal structures, (B) the combined GTP cMD conformers, (C) the combined cMD conformers and (D) the single aMD trajectory conformers. The protein structure of reference corresponds to the RhoA-GDP complex from *Homo Sapiens* (PDB code 1FTN).

The results of applying FTMap and subsequent probe analysis to aMD derived conformers are shown in **Figure 7**. Also shown are the results obtained for the ensemble of crystal structures, a single cMD trajectory and the combined cMD trajectories. The overall similarity of results between the four datasets is clearly apparent. The major hot spots detected in the ensemble of crystal structures are also observed in our three cMD trajectories and single aMD trajectory. For example the Asp-67, Thr-68, Ala-69, Gly-70, Gln-71 cluster in α2. However, in general, probe occupancies are notably higher in the identified distal regions of the aMD conformations. This provides evidence that the accelerated simulation of a single structure can sample more accessible hotspots, by exploring a larger region of the conformational space, than that contained in either our large set of crystal structures or combined cMD trajectories. In addition, some binding pockets observed in the aMD conformations were present at very low or nearly negligible probe occupancy in the crystal or cMD datasets. There is a noticeably higher occupancy in the α3, β5, α3 and Loop 3 regions in **Figure 7D** compared to **Figures 7A** and **7C** reflecting the sampling of more open pocket conformations in these regions. For example the crystal structures (**Figures 7A**) present a binding pocket in the region of α2 facing β1. In the aMD ensemble (**Figure 7D**), a similar binding pocket is also observed in α2, but expanded onto β5 (the opposite direction).

### Targeting Predicted Binding Sites

**Figure 8:**
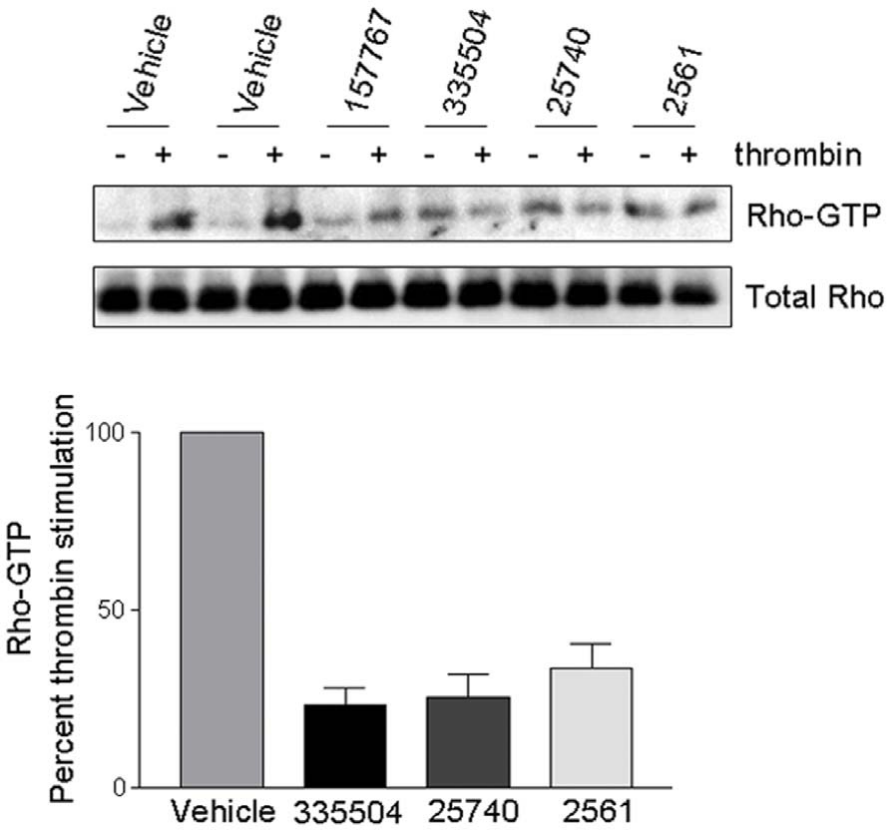
Putative Rho compounds inhibit thrombin stimulated RhoA activation in glioblastoma cells. Compounds were selected from a pocket directed virtual screen and tested on Glioblastoma cells (1321N1). Cells were pre-treated for 1 hr with either DMSO control or 30 µM of compound 157767, 335504, 25740 or 2561 and subsequently stimulated for 15 minutes with either vehicle or 0.5 U/mL thrombin. RhoA activity was assessed via pull-down by rhotekin and total RhoA was determined using an anti-RhoA antibody. Bar graph shows densitometric results from 4 independent experiments. Data are presented as means ± SEM; *P<0.001 vs. vehicle control, one-way ANOVA.

Using the Schrödinger Glide package, [48] compounds from the NCIDS II were docked against the identified α2 and Loop 3 pockets in each structure that reported fragment-binding activity in these regions. Four high-ranking compounds (compound identifiers: 2561, 25740, 157767 and 335504) were selected and their effect on thrombin stimulated RhoA activation in 1321N1 glioblastoma cells assessed (see Methods). Thrombin is a potent mitogen for 1321N1 glioblastoma cells and elicits its cellular responses through activation of PAR-1 receptors, which in turn leads to activation of RhoA. [49], [50], [51] Cells were pre-treated with the compounds at a concentration of 30 µM, subsequently stimulated with thrombin for 15 minutes and RhoA pull down assays were performed to assess increases in GTP-bound RhoA. As shown in **Figure 8**, three of the four compounds (335504, 25740 and 2561) significantly inhibited thrombin-induced RhoA activation by approximately 70–80% relative to vehicle control, while compound 157767 had no significant effect. These inhibitors did not significantly affect resting (unstimulated) levels of active RhoA. These data suggest that computationally selected compounds directed against our novel pockets have the ability to block RhoA activation induced by agonist stimulation in glioblastoma cells.

In summary, our results demonstrate that both cMD and aMD simulations can facilitate the identification of potential small molecule binding sites on Rho proteins. Application of aMD is expected to be particularly valuable when large distributions of crystal structures are not available and when the target protein is known to undergo important conformational changes as a result of its biological activity. Furthermore, the results of virtual screening and subsequent preliminary experimental testing support the hypothesis that distal pockets can potentially interact with small drug-like compounds that may attenuate Rho activity.

### Conclusions

In this work we have conducted an exhaustive search of potential binding pockets of the G-protein Rho, focusing on less conserved binding pockets, distal to the NBS. We have performed our study in an incremental fashion, progressively increasing the sophistication of our methodological approach, whilst reducing the number of analyzed structures. We have proceeded in this way in order to find an optimal compromise between the maximum number of results obtained and the amount of initial information required. To this end, we first sampled the binding site landscape of Rho considering every available crystallographic structure from the RCSB Protein Data Bank, [38] taking into account the three major conformational states revealed by PCA (GTP, GDP and APO). Secondly, we performed cMD simulations on three representative Rho structures, one for each state, and analyzed each simulation for new binding sites. Finally, we analyzed binding sites from an aMD simulation commenced from a single Rho structure.

Our results demonstrate that the binding site landscape of Rho is highly dependent on its conformational state, which is in turn modulated by nucleotide turnover. This necessitates the identification of binding pockets and cognate ligands that account for multiple distinct conformations. Here we have identified a number of novel binding pockets, some of them not present in any resolved crystallographic structure known to date, including sites distal to the NBS and proximal to Loop 3 and Loop 7. In addition, some of these pockets are more accessible in the GDP state, compared with GTP or APO conformations. We postulate that the interaction of drug-like compounds with these pockets may alter the distribution of active and inactive Rho conformations and thereby deregulate Rho’s activity. A more exhaustive virtual screening and rational drug design study, as well as experimental validation is being performed currently to further assess these novel pockets. We note that our preliminary results from these studies are encouraging in that micromolar concentrations of candidate inhibitors directed against these pockets block thrombin stimulated RhoA activation by approximately 70–80% in cell lines. This suggests that the predicted compounds could serve as starting points for lead generation targeted against ligand-induced (or constitutively elevated) Rho activity in cancer cells. We are currently comparing the affinity of these compounds using full dose response curves and have determined that at least for compound 335504, 70% inhibition of thrombin-induced RhoA activation at 10 µM. However, further work is required to definitively characterize the drugability of our predicted sites and compounds.

Finally, we find that dynamic simulations, both conventional and accelerated, can be used as a complementary tool to perform exhaustive binding site identification. Both techniques can enhance sampling of binding pockets, and accelerated MD seems particularly useful given extensive conformational changes. These methods aid in finding the maximal amount of significant binding sites with a minimal amount of input information.

## Materials and Methods

### Crystal Structures Analysis

The Bio3D package [52] was used to query and analyze all available Rho structures in the RCSB Protein Data Bank. [38] Principal component analysis was employed to examine the conformational relationships between superposed crystal structures and simulated conformers. The application of PCA to distributions of experimental structures and molecular dynamics trajectories, along with its ability to provide considerable insight into the nature of conformational differences in a range of protein families has been previously discussed. [53], [54], [55], [56], [57], [58] Briefly, PCA is based on the diagonalization of the covariance matrix, *C*, with elements C_ij_ built from the Cartesian coordinates, *r*, of the superimposed Rho structures (eq. 1):
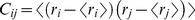
(1)where i and j represent all possible pairs of 3N Cartesian coordinates (where N is the number of atoms) being considered. The eigenvectors of the covariance matrix correspond to a linear basis set of the distribution of structures, referred to as PCs, whereas the eigenvalues provide the variance of the distribution along the corresponding eigenvectors.

### Sequence Conservation Analysis

The PFAM alignment PF00071 was used as a basis for examining sequence conservation within the wider Ras superfamily. [59] To assess the level of conservation at each position in the alignment, the entropy per position was calculated. “Entropy” is based on Shannon’s information entropy for both a 21-letter alphabet (20 amino acids and a gap character) and a seven-letter alphabet (six groups of amino acids and a gap character) [60], [61] (eq. 2):
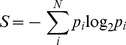
(2)where *S* is Shannon’s entropy, *p_i_* is the frequency of each alphabet’s letter at position *i* and *N* is the alphabet’s size (7 or 21). The six groups chosen were aliphatic (A, V, L, I, M and C), aromatic (F, W, Y and H), polar (S, T, N and Q), positive (K and R), negative (D and E), and finally special conformations (G and P). Entropy scores plotted in **Figure 3** are normalized so that conserved (low entropy) columns score 1 and diverse (high entropy) columns score 0 (eq. 3):
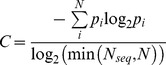
(3)where C is the normalized entropy, *p_i_* is the frequency of each alphabet’s letter at position *i*, *N* is the alphabet’s size and *N_seq_* is the length of the sequence. We define a position to be conserved if the entropy 21 or entropy 7 at a position is >0.6. Positions in which more than 30% of the sequences have gaps were excluded from all sequence conservation analysis.

### Exposed Surface Area

Percent solvent exposure per position was calculated with the NACCESS program. [62] A residue was considered to be exposed when the accessible surface area of the residue was more than 40% of the measured accessible surface area of that residue in an extended Ala-X-Ala tripeptide context.

### Binding Site Mapping

We used the FTMap method of Brenke and co-works to highlight protein surface regions that have the potential to bind the highest number of small molecular probes. [40] Both crystal structures and each cluster representative form cMD and aMD were subject to fragment mapping. Hot-spot residues (those that comprise prominent fragment binding sites) were analyzed across all structures. A residue was assumed to be in contact with a probe molecule (and assigned a probe occupancy value of 1 for a given structure) if any two heavy atoms from the probe and residue were closer than 5.0 Å. The probe occupancy per residue in a given set of structures was obtained by normalizing the summed probe occupancy count for each residue by the number of structures in the input dataset.

We also analyzed crystal structure and trajectory conformers with the SiteMap method from Schrödinger, Inc. New York. [41], [42] SiteMap identifies potential binding sites based on geometric and energetic properties, linking together proximal grid points based on how close the grid points are to the protein surface and how well sheltered they are from solvent. [41], [42] For our purposes, a protein residue from a particular structure was assumed to be part of a potential binding site if a favorable SiteMap grid point was within 5.0 Å of any heavy atom on that residue. The occupancy per residue for a given structure set was then obtained by normalizing the counts for each equivalent residue by the number of structures in the input dataset.

### Molecular Dynamics Simulations

Simulations were performed using the AMBER10 package [63] and corresponding all-atom potential function ff99SB. [64] Operational parameters include periodic boundary conditions, TIP3P water and charge-neutralizing counter ions, with full particle-mesh Ewald electrostatics. A 2fs time step and a 10 Å cutoff were used for the truncation of VDW non-bonded interactions. Constant volume heating (to 300 K) was performed over 10 ps, followed by constant temperature (300 K), constant pressure (1 atm) equilibration for an additional 200 ps. Finally, constant pressure constant temperature production dynamics was performed for both conventional and accelerated MD implementations. The SHAKE algorithm was used to constrain all covalent bonds involving hydrogen atoms. In order to simultaneously enhance the sampling of internal and diffusive degrees of freedom a dual boosting aMD approach was employed, based on separate torsional and total boost potentials. [37], [65] aMD starting structures and standard operational parameters were identical to those used for cMD. The energy level, *E*, below which the boost is applied and tuning parameter, α, that modulates the depth and local roughness of basins in the modified potential, were based on a previous work. [65].

Average-linkage hierarchical clustering based on pairwise RMSD analysis was used to inspect the dominant conformations sampled by each simulation. Inspection of the clustering dendogram was used to partition structures from each trajectory into 24 dominant groups (ranked according to their populations). The closest structure to the average structure from each cluster, in terms of RMSD, was chosen as a representative conformation for further fragment mapping analysis.

### Virtual Screening

Schrödinger’s Glide package [48] was used to screen a subset of the NCIDS II small molecule library [66] against α2 and Loop 3 predicted distal binding pockets. The full NCIDS II (comprising 3881 compounds) was first filtered (to yield a subset of 2291 compounds) by applying the following criteria: molecular weight <553 Da, number of rotatable bonds ≤14, number of potential donor Hydrogen bond atoms ≤6, number of potential acceptor Hydrogen bond atoms ≥3, Hydrophilic polar surface area ≥30 Å, and predicted octanol/water partitioning ≥ −0.5 QlogP. Screening against pocket grids generated for each major conformation in crystallographic and simulated conformers (78 total grids) was performed at the XP level as described previously. [35] Only the compounds with a docking score of −8 or lower were collected and ranked from all grids for each pocket.

### Rho Activation Assay

An affinity pull-down assay using a glutathione S-transferase (GST) fusion protein of the RhoA binding domain of the RhoA effector rhotekin was performed to determine RhoA activation in 1321N1 glioblastoma cells. Cells were lysed in buffer containing 50 mM Tris, pH 7.4, 0.1% Triton X-100, 150 mM NaCl, 5 mM MgCl2 and 10% glycerol, supplemented with protease and phosphatase inhibitors. Cell lysates were clarified by centrifugation and subsequently incubated with the sepharose-bound GST-rhotekin-RhoA binding domain for 50 minutes at 4°C. The beads and the precipitated proteins were washed, boiled and resolved by SDS-PAGE. Total RhoA was detected in the whole cell lysate by immunoblotting with a RhoA antibody (Santa Cruz). GTP-bound RhoA was normalized to total RhoA.

## Supporting Information

Figure S1 **The results of RMSD based clustering of available Rho structures.** Structure labels are colored by nucleotide state (red for GTP, green for GDP, pink for GXP and gray for nucleotide free).
(JPG)

Figure S2 **Binding site characterization with FTMap and SiteMap.** FTMap calculated probe occupancy (black) and SiteMap calculated grid occupancy (gray) per position across all available Rho crystal structures (correlation value, r = 0.9). See main text for further details.
(JPG)

Figure S3 **Binding site characterization in single** ***versus*** **multiple structures.** Fragment probe occupancy per position in a single crystal structure (PDB code 1FTN, gray bars) and averaged across all available crystal structures (black lines, see main text for details).
(JPG)

Figure S4 **Residue-residue plot of correlated motions.** The extent of correlation for all equivalent residue pairs (of Cα atomic displacement) during Rho (lower triangle) and Ras (upper triangle) aMD simulations. The correspondence of Rho and Ras residues was determined from structural alignment with gap positions indicated with a broken axis line (see Ras positions 122–136 that represent a Rho specific insert). The color scale runs from pink (for values ranging between −1 to −0.75), through white (−0.25 to 0.25) to cyan (0.75 to 1). Negative values are indicative of displacements along opposite directions, namely anticorrelated motions, whereas positive values depict correlated motions occurring along the same direction. Major secondary structure elements of Rho are indicated schematically with helices in black and strands in gray. All calculations were were performed with the Bio3D package.
(JPG)

Table S1 Ensemble of resolved Rho crystal structures used in this work.
(DOC)
