# Supplementary figures and images for: Identification of Potential Small Molecule Binding Pockets on Rho Family GTPases
Source: PLoS One. 2012 Jul 16;7(7):e40809. doi: 10.1371/journal.pone.0040809 (PMC3397943; doi:10.1371/journal.pone.0040809)

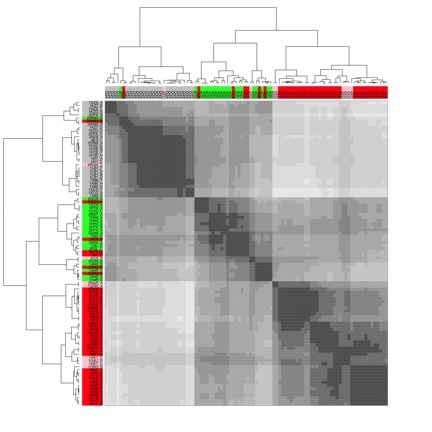

Supplement: Figure S1 — The results of RMSD based clustering of available Rho structures. Structure labels are colored by nucleotide state (red for GTP, green for GDP, pink for GXP and gray for nucleotide free). (JPG) [file pone.0040809.s001.jpg]

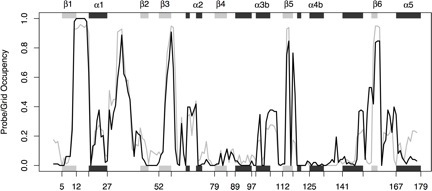

Supplement: Figure S2 — Binding site characterization with FTMap and SiteMap. FTMap calculated probe occupancy (black) and SiteMap calculated grid occupancy (gray) per position across all available Rho crystal structures (correlation value, r = 0.9). See main text for further details. (JPG) [file pone.0040809.s002.jpg]

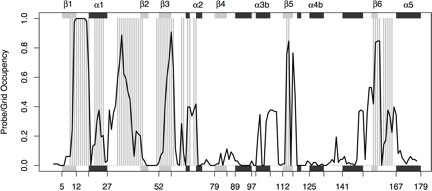

Supplement: Figure S3 — Binding site characterization in single versus multiple structures. Fragment probe occupancy per position in a single crystal structure (PDB code 1FTN, gray bars) and averaged across all available crystal structures (black lines, see main text for details). (JPG) [file pone.0040809.s003.jpg]

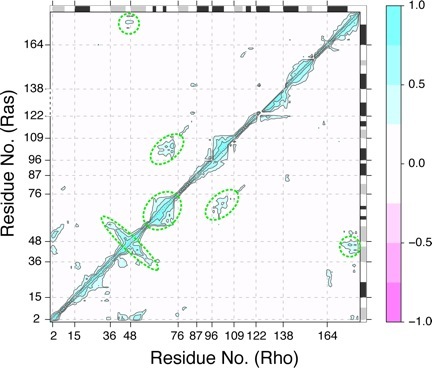

Supplement: Figure S4 — Residue-residue plot of correlated motions. The extent of correlation for all equivalent residue pairs (of Cα atomic displacement) during Rho (lower triangle) and Ras (upper triangle) aMD simulations. The correspondence of Rho and Ras residues was determined from structural alignment with gap positions indicated with a broken axis line (see Ras positions 122–136 that represent a Rho specific insert). The color scale runs from pink (for values ranging between −1 to −0.75), through white (−0.25 to 0.25) to cyan (0.75 to 1). Negative values are indicative of displacements along opposite directions, namely anticorrelated motions, whereas positive values depict correlated motions occurring along the same direction. Major secondary structure elements of Rho are indicated schematically with helices in black and strands in gray. All calculations were were performed with the Bio3D package. (JPG) [file pone.0040809.s004.jpg]
