# Supplementary material for: Identification of Potential Small Molecule Binding Pockets on Rho Family GTPases
Source: PLoS One. 2012 Jul 16;7(7):e40809. doi: 10.1371/journal.pone.0040809 (PMC3397943; doi:10.1371/journal.pone.0040809)
Supplement: Table S1 — Ensemble of resolved Rho crystal structures used in this work. (DOC) [file pone.0040809.s005.doc]

| **PDB ID** | **HETATOMs** | **Resolution (Å)** | **Source** | **Reference** |
| --- | --- | --- | --- | --- |
| 1A2B | GSP | 2.4 | *Homo Sapiens* |  |
| 1A4R | GNH, GDP, MG | 2.5 | *Homo Sapiens* |  |
| 1AN0 | GDP, MG | 2.8 | *Homo Sapiens* |  |
| 1CC0 | GDP, MG | 5 | *Homo Sapiens* |  |
| 1CXZ | GSP, MG | 2.2 | *Homo Sapiens* |  |
| 1DOA | MG, GDP, GER | 2.6 | *Homo Sapiens* |  |
| 1DS6 | GDP, MG | 2.4 | *Homo Sapiens* |  |
| 1E96 | MG, GTP | 2.4 | *Homo Sapiens* |  |
| 1FOE | SO4 | 2.8 | *Homo Sapiens* |  |
| 1FTN | GDP, MG | 2.1 | *Homo Sapiens* |  |
| 1G4U | AF3, GDP, MG | 2.3 | *Homo Sapiens* |  |
| 1GRN | AF3, GDP, MG | 2.1 | *Homo Sapiens* |  |
| 1GWN | MG, GTP | 2.1 | *Mus Musculus* |  |
| 1GZS | SO4 | 2.3 | *Homo Sapiens* |  |
| 1HE1 | NI, GDP, AF3, MG | 2 | *Homo Sapiens* |  |
| 1HH4 | GER, MG, GDP | 2.7 | *Homo Sapiens* |  |
| 1I4D | GDP, MG | 2.5 | *Homo Sapiens* |  |
| 1I4L | GDP, MG | 2.7 | *Homo Sapiens* |  |
| 1I4T | GNP, MG | 2.6 | *Homo Sapiens* |  |
| 1KI1 | SO4 | 2.3 | *Homo Sapiens* |  |
| 1KMQ | DIO, GNP, MG | 1.6 | *Homo Sapiens* |  |
| 1KZ7 |  | 2.4 | *Homo Sapiens* |  |
| 1KZG |  | 2.6 | *Homo Sapiens* |  |
| 1LB1 |  | 2.8 | *Homo Sapiens* |  |
| 1M7B | GTP, MG | 2 | *Homo Sapiens* |  |
| 1MH1 | GNP, MG | 1.4 | *Homo Sapiens* |  |
| 1NF3 | GNP, MG | 2.1 | *Homo Sapiens* |  |
| 1OW3 | MGF, GDP, MG | 1.8 | *Homo Sapiens* |  |
| 1S1C | GNP, MG | 2.6 | *Homo Sapiens* |  |
| 1X86 | PO4 | 3.2 | *Homo Sapiens* |  |
| 1XCG |  | 2.5 | *Homo Sapiens* |  |
| 1Z2C | GNP, MG | 3 | *Homo Sapiens* |  |
| 2ATX | GNP, MG | 2.6 | *Homo Sapiens* |  |
| 2CLS | MG, GTP | 2.3 | *Homo Sapiens* |  |
| 2DFK | GOL, SO4 | 2.1 | *Homo Sapiens* |  |
| 2FJU | GSP, CA, MG | 2.2 | *Homo Sapiens* |  |
| 2G0N | GDP, CL, MG | 1.9 | *Homo Sapiens* |  |
| 2GCN | EDO, GDP, MG | 1.8 | *Homo Sapiens* |  |
| 2GCO | GNP, MG | 1.4 | *Homo Sapiens* |  |
| 2H7V | GDP, MG | 2.6 | *Homo Sapiens* |  |
| 2IC5 | MG, CL, GNP, GDP, BTB | 1.9 | *Homo Sapiens* |  |
| 2NGR | AF3, GDP, MG | 1.9 | *Homo Sapiens* |  |
| 2NZ8 |  | 2 | *Homo Sapiens* |  |
| 2ODB | CL, MG, SO4, GCP | 2.4 | *Homo Sapiens* |  |
| 2OV2 | MG, CL, GCP, EDO | 2.1 | *Homo Sapiens* |  |
| 2P2L | GDP, ZN | 1.9 | *Homo Sapiens* |  |
| 2QME | GOL, GCP, MG | 1.8 | *Homo Sapiens* |  |
| 2QRZ | GCP, SO4, MG | 2.4 | *Homo Sapiens* |  |
| 2RGN | GDP, MG, ALF | 3.5 | *Homo Sapiens* |  |
| 2V55 | SO4, GTP, MG, ANP | 3.7 | *Homo Sapiens* |  |
| 2VRW | ZN | 1.8 | *Homo Sapiens* |  |
| 2W2T | GDP, MG | 2 | *Homo Sapiens* |  |
| 2W2X | MG, GSP | 2.3 | *Homo Sapiens* |  |
| 2WM9 | GOL | 2.2 | *Homo Sapiens* |  |
| 2WMN | GDP | 2.4 | *Homo Sapiens* |  |
| 2WMO | MG, GTP | 2.2 | *Homo Sapiens* |  |
| 3A58 | PO4, GNP, MG | 2.6 | *Saccharomyces Cerevisiae* |  |
| 3BJI | ZN | 2.6 | *Homo Sapiens* |  |
| 3EG5 | MG, GNP | 2.7 | *Mus Musculus* |  |

**Supplementary References**

1. Ihara K, Muraguchi S, Kato M, Shimizu T, Shirakawa M, et al. (1998) Crystal structure of human RhoA in a dominantly active form complexed with a GTP analogue. J. Biol. Chem. 273: 9656-9666.

2. Rudolph MG, Wittinghofer A, Vetter IR (1999) Nucleotide binding to the G12V-mutant of Cdc42 investigated by X-ray diffraction and fluorescence spectroscopy: two different nucleotide states in one crystal. Prot. Sci. 8: 778-787.

3. Kongsaeree P, Cerione RA, Clardy JC To be published.

4. Longenecker K, Read P, Derewenda U, Dauter Z, Liu X, et al. (1999) How RhoGDI binds Rho. Acta Crystallogr. D Biol. Crystallogr. 55: 1503-1515.

5. Maesaki R, Ihara K, Shimizu T, Kuroda S, Kaibuchi K, et al. (1999) The structural basis of Rho effector recognition revealed by the crystal structure of human RhoA complexed with the effector domain of PKN/PRK1. Mol. Cell 4: 793-803.

6. Hoffman GR, Nassar N, Cerione RA (2000) Structure of the Rho family GTP-binding protein Cdc42 in complex with the multifunctional regulator RhoGDI. Cell 100: 345-356.

7. Scheffzek K, Stephan I, Jensen ON, Illenberger D, Gierschik P (2000) The Rac-RhoGDI complex and the structural basis for the regulation of Rho proteins by RhoGDI. Nat. Struct. Biol. 7: 122-126.

8. Lapouge K, Smith SJ, Walker PA, Gamblin SJ, Smerdon SJ, et al. (2000) Structure of the TPR domain of p67phox in complex with Rac.GTP. Mol. Cell 6: 899-907.

9. Worthylake DK, Rossman KL, Sondek J (2000) Crystal structure of Rac1 in complex with the guanine nucleotide exchange region of Tiam1. Nature 408: 682-688.

10. Wei Y, Zhang Y, Derewenda U, Liu X, Minor W, et al. (1997) Crystal structure of RhoA-GDP and its functional implications. Nat. Struct. Biol. 4: 699-703.

11. Stebbins CE, Galan JE (2000) Modulation of host signaling by a bacterial mimic: structure of the Salmonella effector SptP bound to Rac1. Mol. Cell 6: 1449-1460.

12. Nassar N, Hoffman GR, Manor D, Clardy JC, Cerione RA (1998) Structures of Cdc42 bound to the active and catalytically compromised forms of Cdc42GAP. Nat. Struct. Biol. 5: 1047-1052.

13. Garavini H, Riento K, Phelan JP, McAlister MS, Ridley AJ, et al. (2002) Crystal structure of the core domain of RhoE/Rnd3: a constitutively activated small G protein. Biochem. 41: 6303-6310.

14. Buchwald G, Friebel A, Galan JE, Hardt WD, Wittinghofer A, et al. (2002) Structural basis for the reversible activation of a Rho protein by the bacterial toxin SopE. Embo J. 21: 3286-3295.

15. Wurtele M, Wolf E, Pederson KJ, Buchwald G, Ahmadian MR, et al. (2001) How the Pseudomonas aeruginosa ExoS toxin downregulates Rac. Nat. Struct. Biol. 8: 23-26.

16. Grizot S, Faure J, Fieschi F, Vignais PV, Dagher MC, et al. (2001) Crystal structure of the Rac1-RhoGDI complex involved in nadph oxidase activation. Biochem. 40: 10007-10013.

17. Tarricone C, Xiao B, Justin N, Walker PA, Rittinger K, et al. (2001) The structural basis of Arfaptin-mediated cross-talk between Rac and Arf signalling pathways. Nature 411: 215-219.

18. Snyder JT, Worthylake DK, Rossman KL, Betts L, Pruitt WM, et al. (2002) Structural basis for the selective activation of Rho GTPases by Dbl exchange factors. Nat. Struct. Biol. 9: 468-475.

19. Longenecker K, Read P, Lin SK, Somlyo AP, Nakamoto RK, et al. (2003) Structure of a constitutively activated RhoA mutant (Q63L) at 1.55 A resolution. Acta Crystallogr. D Biol. Crystallogr. 59: 876-880.

20. Rossman KL, Worthylake DK, Snyder JT, Siderovski DP, Campbell SL, et al. (2002) A crystallographic view of interactions between Dbs and Cdc42: PH domain-assisted guanine nucleotide exchange. Embo J. 21: 1315-1326.

21. Fiegen D, Blumenstein L, Stege P, Vetter IR, Ahmadian MR (2002) Crystal structure of Rnd3/RhoE: functional implications. Febs. Letters 525: 100-104.

22. Hirshberg M, Stockley RW, Dodson G, Webb MR (1997) The crystal structure of human rac1, a member of the rho-family complexed with a GTP analogue. Nat. Struct. Biol. 4: 147-152.

23. Garrard SM, Capaldo CT, Gao L, Rosen MK, Macara IG, et al. (2003) Structure of Cdc42 in a complex with the GTPase-binding domain of the cell polarity protein, Par6. Embo J. 22: 1125-1133.

24. Graham DL, Lowe PN, Grime GW, Marsh M, Rittinger K, et al. (2002) MgF(3)(-) as a transition state analog of phosphoryl transfer. Chem. & Biol. 9: 375-381.

25. Dvorsky R, Blumenstein L, Vetter IR, Ahmadian MR (2004) Structural insights into the interaction of ROCKI with the switch regions of RhoA. J Biol. Chem. 279: 7098-7104.

26. Kristelly R, Gao G, Tesmer JJ (2004) Structural determinants of RhoA binding and nucleotide exchange in leukemia-associated Rho guanine-nucleotide exchange factor. J. Biol. Chem. 279: 47352-47362.

27. Derewenda U, Oleksy A, Stevenson AS, Korczynska J, Dauter Z, et al. (2004) The crystal structure of RhoA in complex with the DH/PH fragment of PDZRhoGEF, an activator of the Ca(2+) sensitization pathway in smooth muscle. Structure 12: 1955-1965.

28. Rose R, Weyand M, Lammers M, Ishizaki T, Ahmadian MR, et al. (2005) Structural and mechanistic insights into the interaction between Rho and mammalian Dia. Nature 435: 513-518.

29. Hemsath L, Dvorsky R, Fiegen D, Carlier MF, Ahmadian MR (2005) An electrostatic steering mechanism of Cdc42 recognition by Wiskott-Aldrich syndrome proteins. Mol. Cell 20: 313-324.

30. Pike ACW, Yang X, Colebrook S, Gileadi O, Sobott F, et al. To be published.

31. Xiang S, Kim EY, Connelly JJ, Nassar N, Kirsch J, et al. (2006) The crystal structure of Cdc42 in complex with collybistin II, a gephyrin-interacting guanine nucleotide exchange factor. J. of Mol. Biol. 359: 35-46.

32. Jezyk MR, Snyder JT, Gershberg S, Worthylake DK, Harden TK, et al. (2006) Crystal structure of Rac1 bound to its effector phospholipase C-beta2. Nat. Struct. Mol. Biol. 13: 1135-1140.

33. Ugochukwu E, Yang X, Zao Y, Elkins J, Gileadi C, et al. To be published.

34. Dias SM, Cerione RA (2007) X-ray crystal structures reveal two activated states for RhoC. Biochem. 46: 6547-6558.

35. Prehna G, Ivanov MI, Bliska JB, Stebbins CE (2006) Yersinia virulence depends on mimicry of host Rho-family nucleotide dissociation inhibitors. Cell 126: 869-880.

36. Yang X, Ugochukwu E, Elkins J, Doyle D To be published.

37. Chhatriwala MK, Betts L, Worthylake DK, Sondek J (2007) The DH and PH domains of Trio coordinately engage Rho GTPases for their efficient activation. J. Mol. Biol. 368: 1307-1320.

38. Yang X, Ugochukwu E, Elkins J, Soundararajan M, Eswaran J, et al. To be published.

39. Ugochukwu E, Yang X, Elkins JM, Burgess-Brown N, Knapp S, et al. To be published.

40. Prehna G, Stebbins CE (2007) A Rac1-GDP trimer complex binds zinc with tetrahedral and octahedral coordination, displacing magnesium. Acta Crystallogr. D Biol. Crystallogr. 63: 628-635.

41. Phillips MJ, Calero G, Chan B, Ramachandran S, Cerione RA (2008) Effector proteins exert an important influence on the signaling-active state of the small GTPase Cdc42. J. Biol. Chem. 283: 14153-14164.

42. Lutz S, Shankaranarayanan A, Coco C, Ridilla M, Nance MR, et al. (2007) Structure of Galphaq-p63RhoGEF-RhoA complex reveals a pathway for the activation of RhoA by GPCRs. Science 318: 1923-1927.

43. Komander D, Garg R, Wan PT, Ridley AJ, Barford D (2008) Mechanism of multi-site phosphorylation from a ROCK-I:RhoE complex structure. Embo J. 27: 3175-3185.

44. Rapley J, Tybulewicz VL, Rittinger K (2008) Crucial structural role for the PH and C1 domains of the Vav1 exchange factor. EMBO Rep 9: 655-661.

45. Bunney TD, Opaleye O, Roe SM, Vatter P, Baxendale RW, et al. (2009) Structural insights into formation of an active signaling complex between Rac and phospholipase C gamma 2. Mol. Cell 34: 223-233.

46. Yang J, Zhang Z, Roe SM, Marshall CJ, Barford D (2009) Activation of Rho GTPases by DOCK exchange factors is mediated by a nucleotide sensor. Science 325: 1398-1402.

47. Yamashita M, Kurokawa K, Sato Y, Yamagata A, Mimura H, et al. (2010) Structural basis for the Rho- and phosphoinositide-dependent localization of the exocyst subunit Sec3. Nat. Struct. Mol. Biol. 17: 180-186.

48. Chrencik JE, Brooun A, Zhang H, Mathews, II, Hura GL, et al. (2008) Structural basis of guanine nucleotide exchange mediated by the T-cell essential Vav1. J. Mol. Biol. 380: 828-843.

49. Lammers M, Meyer S, Kuhlmann D, Wittinghofer A (2008) Specificity of interactions between mDia isoforms and Rho proteins. J. Biol. Chem. 283: 35236-35246.
